# Mortality among ischemic and nonischemic heart failure patients with a primary implantable cardioverter‐defibrillator

**DOI:** 10.1002/joa3.12651

**Published:** 2021-10-29

**Authors:** Pil‐Sung Yang, Younghyun Kang, Han‐Joon Bae, Jung‐Hoon Sung, Hyung‐Deuk Park, Boyoung Joung

**Affiliations:** ^1^ Department of Cardiology CHA Bundang Medical Center CHA University Seongnam Republic of Korea; ^2^ Medtronic Korea Ltd. Seoul Republic of Korea; ^3^ Division of Cardiology Department of Internal Medicine Daegu Catholic University Medical Center Daegu Republic of Korea; ^4^ Division of Cardiology Department of Internal Medicine Severance Cardiovascular Hospital Yonsei University College of Medicine Seoul Republic of Korea

**Keywords:** defibrillator, heart failure, ischemic, mortality, nonischemic

## Abstract

**Background:**

The efficacy of implantable cardioverter defibrillators (ICDs) for primary prevention is controversial in patients with nonischemic heart failure (HF). We evaluated the mortality and predictors of mortality in patients with prophylactic ICD implantation for ischemic and nonischemic HF.

**Methods:**

From 2008 to 2017, 1097 patients (667, nonischemic HF and 430, ischemic HF) who underwent prophylactic ICD implantation, were identified from the Korean National Health Insurance Service database. We used propensity score overlap weighting to correct the differences between two groups.

**Results:**

Those with ischemic HF were older (67.0 ± 10.1 vs 61.8 ± 14.2 years), more often male (71.4% vs 63.7%), and had more comorbidities than patients with nonischemic HF. During a median follow‐up of 37.3 months (interquartile range [IQR], 14.2‐53.8 months), all‐cause mortality was higher in unweighted patients with ischemic HF than in those with nonischemic HF (10.9 vs 6.4 per 100 person‐years; hazard ratio [HR], 1.74; 95% confidence interval [CI], 1.38‐2.20; *P* < .001). However, after weighting, the annual all‐cause mortality rate was similar in both groups (9.5 vs 8.8 per 100 person‐years), with no significant difference in the risk of all‐cause mortality (HR, 1.08; 95% CI, 0.68‐1.71; *P* = .755). Older age and chronic kidney disease were independent predictors of all‐cause mortality in both groups. There was no significant difference in cardiac and noncardiac mortality between the weighted nonischemic and ischemic HF groups.

**Conclusions:**

The all‐cause, cardiac, and noncardiac mortality rates were similar between patients with nonischemic and ischemic HF who underwent prophylactic ICD implantation.

## Graphical abstract

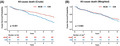

## 1 INTRODUCTION

As implantable cardioverter defibrillators (ICDs) therapy reduces morbidity and mortality as a part of the primary prevention strategy in patients with heart failure with reduced left ventricular ejection fraction (HFrEF),^1^, ^2^, ^3^, ^4^ prophylactic ICD therapy for the primary prevention of sudden cardiac death (SCD) is recommended to reduce mortality in select patients with HFrEF with an left ventricular ejection fraction (LVEF) ≤35%.^5^, ^6^

Evidence for the benefit of prophylactic ICD therapy is much stronger for patients with ischemic heart failure (HF) than for patients with nonischemic HF.^2^, ^7^ No single study based exclusively on patients with HFrEF unrelated to coronary artery disease has demonstrated a reduction in mortality because of ICD implantation. Moreover, many trials showing the benefit of prophylactic ICD therapy have been studied in patients enrolled more than 20 years ago, and might not reflect the current patient characteristics and current management of HFrEF. Indeed, recent advances have affected the risk profile of patients with HFrEF, leading to a 44% reduction in SCD risk over the past two decades.^8^, ^9^ Therefore, the beneficial prognostic effects of ICDs might be different because of the improved risk profile. The Danish Study to Assess the Efficacy of ICDs in Patients with Non‐ischemic Systolic Heart Failure on Mortality (DANISH) trial questioned the efficacy of primary prophylactic ICD therapy in patients with nonischemic cardiomyopathy combined with contemporary treatments.^10^ This trial showed that ICD implantation did not significantly decrease the rates of all‐cause death in patients with nonischemic HF, even though the occurrence of SCD was effectively reduced.^10^

The addition of cardiac resynchronization therapy (CRT) to ICD implantation (58% of patients in both arms of the DANISH trial carried CRT devices) not only modifies the possibility of improving left ventricular ejection fraction, especially in nonischemic cases but can also reduce the morbidity and mortality outcomes.^11^, ^12^, ^13^, ^14^ Pooled data from previous meta‐analyses demonstrated that even after the elimination of CRT trials, ICD‐only therapy accomplished a reduction in total mortality, ranging between 26% and 31% in patients with nonischemic HF.^15^, ^16^

The present study aimed to assess whether the rates of all‐cause, cardiac, and noncardiac mortality differed between HFrEF patients with nonischemic and ischemic HF after ICD implantation as a primary prevention strategy. We aimed to identify the specific predictors of mortality in ischemic and nonischemic HF populations and to evaluate the rates of the other causes of death.

## 2 METHODS

### 2.1 Data source

The majority (97.1%) of the Korean population is mandatorily subscribed to the National Health Insurance Service (NHIS), a single insurer managed by the Korean government, with the remaining 3% categorized as medical aid subjects. As the database also includes information on the medical aid population, it can be considered to be representative of the entire Korean population.^17^, ^18^, ^19^, ^20^ All pertinent data, including patients’ sociodemographic information, data on the use of inpatient and outpatient services, pharmacy‐dispensing claims, and mortality rate data, can be accessed through this database. The NHIS database can be accessed only through the wired network at the designated analysis center, with formal payment according to the period of browsing and analyzing the data and applying strict regulations regarding data release (https://nhiss.nhis.or.kr/).

### 2.2 Study population

The study cohort consisted of all admissions that included a procedure for ICDs (procedure codes: O2008, O0211, and O0212) from the entire Korean population in the Korean NHIS database. In accordance with the data provision policy of the Korea NHIS, 50% random sampling was performed when the study cohort was formed. Primary prevention ICD was defined by exclusion of patients with a previous history of sudden cardiac arrest or sustained ventricular tachycardia. After excluding patients with a previous history of sudden cardiac arrest or sustained ventricular tachycardia, 1,097 patients aged 19 years or older with HF who received primary prevention ICD therapy were identified from January 1, 2008 to December 31, 2017.

In Korea, ICD implantation is strictly managed by the Korean NHIS, so all HF patients who underwent primary prevention ICD are subject to one of the following criteria. In patients with ischemic HF, (1) LVEF ≤30%, (2) LVEF of 31%–35% with symptoms of NYHA class II, III, (3) LVEF ≤40% with nonsustained ventricular tachycardia and hemodynamically significant ventricular fibrillation or sustained ventricular tachycardia is induced in the electrophysiologic study. In patients with nonischemic HF, (4) patients with NYHA class II, III symptom, and LVEF ≤35% despite adequate medical treatment for 3 months or longer. Therefore, all patients with primary prevention ICD in this study are HFrEF patients with LVEF ≤40% except in very few cases where the Korean NHIS regulations are violated.

Ischemic HF was defined by a previous hospitalization for myocardial infarction diagnosed by coronary angiography, previous diagnosis of ischemic cardiomyopathy (ICD‐10 code: I25.5), or previous history of percutaneous coronary intervention or coronary artery bypass surgery. After classifying the ischemic HF, the remaining patients were classified as having nonischemic HF.

### 2.3 Outcomes

The primary clinical outcome was all‐cause mortality. Data on vital status and date of death were confirmed from the National Population Registry of the Korea National Statistical Office using a unique personal identification number, in which central registration of death was conducted on the basis of the death certificates.^17^, ^18^, ^19^, ^20^, ^21^, ^22^ This approach provides a complete event ascertainment given that the NHIS and National Statistical Office are national organizations that cover all Korean subjects.

We also assessed cardiac death, noncardiac death, and arrhythmic death as secondary outcomes. Cause‐specific mortality was analyzed based on the causes of death confirmed by the Korea National Statistical Office. Cardiac death included death related to HF and coronary disease, sudden and other cardiac deaths, and noncardiac death included death related to cancer, cerebrovascular disease, and other causes. The definitions of the clinical outcomes are presented in Table S1.

### 2.4 Statistical analysis

The baseline characteristics of participants with nonischemic and ischemic HF were compared using the Student's *t* test and Pearson's chi‐square test. Simple between‐group analyses were conducted using the Student's *t* test. Categorical variables were compared using Fisher's exact test.

We used an overlap weighting approach based on propensity scores to allow an unbiased comparison in the main analyses. Propensity scores were calculated using the following variables: age; gender; economic status; history of atrial fibrillation; hypertension; peripheral artery disease; chronic kidney disease; chronic obstructive pulmonary disease; liver disease; cancer; and treatment with aspirin, P_2_Y_12_ inhibitors, oral anticoagulants, aldosterone antagonists, furosemide, beta‐blockers, angiotensin‐converting enzyme inhibitors (ACEIs), angiotensin receptor blockers (ARBs), and the anti‐arrhythmic drugs digoxin and statin. The overlap weight was calculated as 1 minus the propensity score for the ischemic HF patients, and the propensity score for the nonischemic HF patients.^23^ The balance between the populations was evaluated by standardized differences of all baseline covariates using a threshold of 0.1 to indicate imbalance.

The incidence of events was calculated by dividing the number of events by the person‐times at risk, with 95% confidence intervals (CI) estimated by exact Poisson distributions. The incidence of death was compared using the weighted log‐rank test, and the weighted failure curves were plotted. Cox proportional hazards regression was used to compare the nonischemic and ischemic HF groups.

Kaplan‐Meier curves were constructed to estimate the event‐free outcomes in the two study groups using the log‐rank test. A Cox proportional hazards regression model was used to estimate the hazard ratios (HRs) for clinical events. All covariates that reached a significance level of *P* < .1 were included in the multivariate regression model. Statistical significance was set at *P* < .05. Statistical analyses were conducted using SAS version 9.4 (SAS Institute) and R version 4.0.1 (The R Foundation, www.R‐project.org).

## 3 RESULTS

### 3.1 Patient characteristics

From 2008 to 2017, 1097 patients aged 19 years or older who underwent prophylactic ICD implantation were identified from the Korean NHIS database. Those with ischemic HF were older (67.0 ± 10.1 years vs 61.8 ± 14.2), more often male (71.4% vs 63.7%), and had more comorbidities, including hypertension, diabetes, and atrial fibrillation, than patients with nonischemic HF. Moreover, antiplatelet agents, diuretics, beta‐blockers, ACEIs/ARBs, and statins were more frequently used in patients with ischemic HF than in those with nonischemic HF. CRT‐D was implanted in 45.6% and 40.0% of the nonischemic and ischemic HF populations, respectively. The median follow‐up duration was slightly shorter in patients with ischemic HF than in those with nonischemic HF (29.2 months vs 33.0 months, *P* = .010). After weighting, all baseline characteristics were similar between the two groups (Table 1).

**TABLE 1:** Baseline characteristics of patients with nonischemic and ischemic HF who underwent prophylactic ICD implantation

	Crude			Weighted		
	Nonischemic HF (n = 667)	Ischemic HF (n = 430)	SMD	Nonischemic HF (n = 667)	Ischemic HF (n = 430)	SMD
Demographic						
Male	63.7%	71.4%	0.165	64.3%	64.3%	<0.001
Age, years (mean ±SD)	61.8 ± 14.2	67.0 ± 10.1	0.420	66.9 ± 11.0	66.9 ± 10.1	<0.001
Economic status^a^ (mean ±SD)	12.7 ± 5.5	12.6 ± 5.5	0.012	13.0 ± 5.6	13.0 ± 5.3	<0.001
Implanted device			0.113			<0.001
CRT‐D	45.6%	40.0%		50.1%	50.1%	
Dual chamber ICD	23.2%	25.3%		20.8%	20.8%	
Single chamber ICD	31.2%	34.7%		29.1%	29.1%	
Comorbidities						
Hypertension	91.8%	98.6%	0.324	97.8%	97.8%	<0.001
Diabetes	62.4%	85.6%	0.549	81.2%	81.2%	<0.001
Atrial fibrillation	31.9%	36.3%	0.092	36.6%	36.6%	<0.001
Peripheral artery disease	11.8%	23.5%	0.309	19.6%	19.6%	<0.001
Chronic kidney disease	12.6%	20.2%	0.207	14.8%	14.8%	<0.001
COPD	37.6%	45.1%	0.152	45.8%	45.8%	<0.001
Liver disease	24.0%	30.9%	0.156	27.9%	27.9%	<0.001
History of cancer	15.3%	18.6%	0.088	16.8%	16.8%	<0.001
Medications						
Aspirin	53.4%	84.9%	0.581	74.7%	74.7%	<0.001
P_2_Y_12_ inhibitor	13.9%	66.7%	1.008	39.2%	39.2%	<0.001
Oral anticoagulants	24.0%	25.3%	0.018	24.6%	24.6%	<0.001
Aldosterone antagonists	58.0%	67.4%	0.129	64.1%	64.1%	<0.001
Furosemide	61.3%	74.9%	0.167	67.5%	67.5%	<0.001
Beta‐blockers	48.1%	53.7%	0.112	46.0%	46.0%	<0.001
ACEI or ARB	73.3%	80.0%	0.216	77.5%	77.5%	<0.001
Class Ic AAD	1.2%	0.7%	0.078	1.3%	1.3%	<0.001
Class III AAD	19.3%	19.1%	0.165	20.9%	20.9%	<0.001
Digoxin	36.4%	38.8%	0.420	40.5%	40.5%	<0.001
Statins	32.2%	58.6%	0.012	44.4%	44.4%	<0.001

Abbreviation: AAD, Anti‐arrhythmic drugs; ACEI, Angiotensin‐converting enzyme inhibitor; ARB, Angiotensin II receptor blocker; COPD, Chronic obstructive pulmonary disease; CRT‐D, Cardiac resynchronization therapy with defibrillator; HF, Heart failure; ICD, Implantable cardioverter defibrillator; SD, Standard deviation; SMD, Standardized mean difference.

^a^ The economic status was determined on the basis of the relative economic levels categorized into 20 levels according to their health insurance premiums in the index year.

### 3.2 All‐cause death and predictors associated with mortality

**FIGURE 1:**
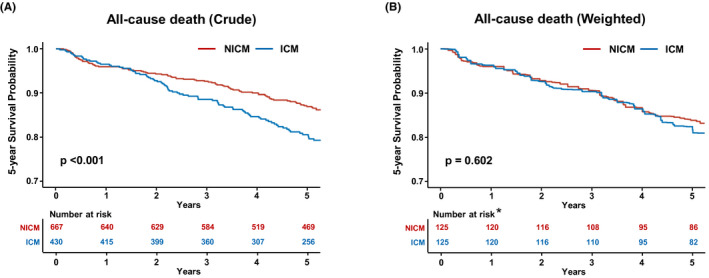
Kaplan‐Meier estimates for survival free from all‐cause death in patients with nonischemic (NICM) and ischemic (ICM) heart failure. A, Crude patients; B, Weighted patients. *The at‐risk table of weighted patients shows the number of patients at risk in the weighted pseudo‐population

During a median follow‐up of 37.3 months (interquartile range [IQR], 14.2‐53.8), 287 patients died (26.2%), resulting in an overall all‐cause mortality rate of 7.9% per 100 person‐years. A total of 151 (of 667) and 136 (of 430) patients died in the nonischemic and ischemic HF groups, respectively. Causes of death showed in Table S2. There were no significant differences in the proportions of cardiac death and noncardiac death between the nonischemic HF and ischemic HF groups. The mortality rate was higher in patients with ischemic HF than in those with nonischemic HF (*P* < .001), with annualized all‐cause mortality rates of 10.9 and 6.4 per 100 person‐years, respectively. Ischemic HF was associated with a 74% higher risk of all‐cause death compared with nonischemic HF (HR, 1.74; 95% CI, 1.38‐2.20, *P* < .001) (Table 2). The Kaplan‐Meier estimate of survival free from all‐cause death was significantly lower in patients with ischemic HF than in those with nonischemic HF (log‐rank *P* < .001; Figure 1A). However, in overlap weighted nonischemic and ischemic HF groups, the annual mortality rate was 8.8 and 9.5 per 100 person‐years, respectively, with no significant difference in the risk of all‐cause death (HR, 1.08; 95% CI, 0.68‐1.71; *P* = .755) (Table 2). The Kaplan‐Meier estimate of survival free from all‐cause death also showed no significant difference between patients with ischemic HF and those with nonischemic HF (log‐rank *P* = .622, Figure 1B); this trend was consistently observed at 1‐ and 5‐year follow‐ups (Table 2).

**TABLE 2:** Incidence rate of all‐cause death in patients with nonischemic and ischemic HF who underwent prophylactic ICD implantation

	Nonischemic HF		Ischemic HF		Absolute difference in event rate (95% CI)	Hazard ratio (95% CI)	*P*‐value
	Number of events	Event rate (/100 person‐years)	Number of events	Event rate (/100 person‐years)			
Crude							
1‐year follow‐up	46	7.2	45	11.0	3.79 (−0.03 to 7.61)	1.53 (1.01 to 2.30)	.043
5‐year follow‐up	137	6.7	125	10.8	4.18 (1.94 to 6.39)	1.62 (1.27 to 2.06)	<.001
Overall	151	6.4	136	10.9	4.49 (2.40 to 6.58)	1.74 (1.38 to 2.20)	<.001
Weighted							
1‐year follow‐up	11	9.0	11	9.6	0.66 (−7.08 to 8.41)	1.07 (0.47 to 2.47)	.870
5‐year follow‐up	32	8.8	35	9.6	0.80 (−3.60 to 5.21)	1.09 (0.68 to 1.76)	.720
Overall	35	8.8	37	9.5	0.70 (−3.48 to 4.89)	1.08 (0.68 to 1.71)	.755

Abbreviations: CI, Confidence interval; HF, Heart failure, ICD; Implantable cardioverter defibrillator.

In multivariate analysis, the factors associated with mortality in patients with nonischemic HF were hypertension (HR, 5.11; 95% CI, 1.56‐16.7; *P* = .007), chronic kidney disease (HR, 1.56; 95% CI, 1.00‐2.44; *P* = .048), and older age (per 10‐year increase) (HR, 1.52; 95% CI, 1.28‐1.80; *P* < .001). The factors associated with mortality in patients with ischemic HF were chronic kidney disease (HR, 2.21; 95% CI, 1.49‐3.29; *P* < .001) and older age (per 10‐year increase) (HR, 1.42; 95% CI, 1.15‐1.77; *P* = .001) (Table 3).

**TABLE 3:** Predictors of all‐cause death in patients with nonischemic and ischemic HF who underwent prophylactic ICD implantation

	Nonischemic HF		Ischemic HF	
	Hazard ratio (95% CI)	*P*‐value	Hazard ratio (95% CI)	*P*‐value
Age (per 10 year increase)	1.52 (1.28‐1.80)	<.001	1.42 (1.15‐1.77)	.001
Chronic kidney disease	1.56 (1.00‐2.44)	.048	2.21 (1.49‐3.29)	<.001
Hypertension	5.11 (1.56‐16.7)	.007	1.65 (0.21‐12.7)	.631
Female	0.85 (0.60‐1.21)	.362	0.79 (0.54‐1.16)	.232
Atrial fibrillation	1.29 (0.91‐1.83)	.154	0.98 (0.69‐1.41)	.926
Diabetes	0.93 (0.64‐1.35)	.703	1.74 (0.90‐3.35)	.100
COPD	1.25 (0.89‐1.76)	.197	1.26 (0.88‐1.81)	.205
History of cancer	1.50 (0.99‐2.27)	.057	1.40 (0.90‐2.19)	.137
Liver disease	1.42 (0.98‐2.06)	.062	0.81 (0.55‐1.20)	.292
Dual chamber (vs single chamber)	1.05 (0.67‐1.63)	.842	1.46 (0.91‐2.35)	.116
CRT‐D (vs ICD only)	0.75 (0.50‐1.12)	.163	1.27 (0.83‐1.96)	.277

Abbreviations: CI, Confidence interval; COPD, Chronic obstructive pulmonary disease; CRT‐D, Cardiac resynchronization therapy with a defibrillator; HF, Heart failure; ICD, Implantable cardioverter defibrillator.

### 3.3 Cardiac, noncardiac, and arrhythmic death

**FIGURE 2:**
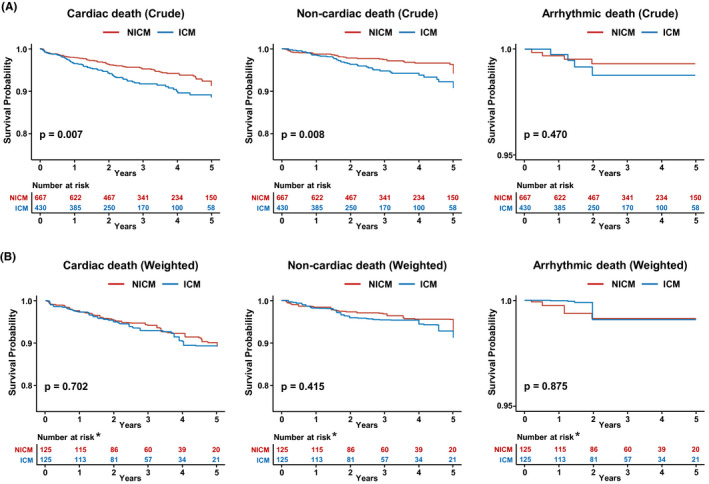
Kaplan‐Meier estimates for survival free from cardiac death (left panels), noncardiac death (middle panels) and arrhythmic death (right panels) in patients with nonischemic (NICM) and ischemic (ICM) HF. A, Crude patients; B, Weighted patients. *The at‐risk table of weighted patients shows the number of patients at risk in the weighted pseudo‐population

Among the causes of death, 140 (48.8%) patients died from cardiovascular causes, and 83 (28.9%) died from noncardiovascular causes. Cardiac (5.3/3.1 per 100 person‐years; HR, 1.65; 95% CI, 1.18‐2.31; *P* = .003) and noncardiac mortalities (3.3 and 1.8 per 100 person‐years; HR, 1.91; 95% CI, 1.24‐2.95; *P* = .004) were also higher in unweighted patients with ischemic HF than in those with nonischemic HF (Table 4). Moreover, the Kaplan‐Meier estimate of survival free from cardiac and noncardiac death was significantly lower in patients with ischemic HF than in those with nonischemic HF (all log‐rank *P* < .05; Figure 2A). However, there was no significant difference in cardiac (4.1 and 4.6 per 100 person‐years) and noncardiac mortality (2.4 and 3.1 per 100 person‐years) between the weighted nonischemic and ischemic HF groups. Furthermore, the rate of arrhythmic death did not differ significantly between the two groups before and after weighting (Table 4). The Kaplan‐Meier estimate of survival free from cardiac, noncardiac, and arrhythmic death also showed no significant difference between the weighted ischemic HF and nonischemic HF groups (Figure 2B).

**TABLE 4:** Incidence rates of cardiac, noncardiac, and arrhythmic deaths in patients with nonischemic and ischemic HF who underwent prophylactic ICD implantation

	Nonischemic HF		Ischemic HF		Absolute difference in event rate (95% CI)	Hazard ratio (95% CI)	*P*‐value
	Number of events	Event rate (100 person‐years)	Number of events	Event rate (100 person‐years)			
Crude							
Cardiac death	74	3.1	66	5.3	2.15 (0.69 to 3.61)	1.65 (1.18 to 2.31)	.003
Noncardiac death	42	1.8	41	3.3	1.50 (0.37 to 2.64)	1.91 (1.24 to 2.95)	.004
Arrhythmic death	4	0.2	4	0.3	0.15 (−0.20 to 0.50)	1.71 (0.42 to 6.87)	.445
Weighted							
Cardiac death	17	4.1	18	4.6	0.42 (−2.48 to 3.32)	1.07 (0.55 to 2.09)	.824
Noncardiac death	10	2.4	12	3.1	0.70 (−1.59 to 2.98)	1.03 (0.55 to 3.06)	.583
Arrhythmic death	1	0.2	1	0.2	‐0.02 (−0.66 to 0.61)	0.88 (0.04 to 18.2)	.936

Abbreviations: CI, Confidence interval; HF, Heart failure; ICD, Implantable cardioverter defibrillator.

## 4 DISCUSSION

The main findings of this study were as follows. In HF patients who underwent prophylactic ICD therapy, patients with ischemic HF were older and had more comorbidities than those with nonischemic HF. All‐cause, cardiac, and noncardiac mortality rates were higher in unweighted patients with ischemic HF than in those with nonischemic HF; however, these rates were similar between the two groups after weighting. The predictors of all‐cause mortality were older age and chronic kidney disease for both ischemic and nonischemic HF patients, whereas hypertension was only associated with death in patients with nonischemic HF. Furthermore, the rates of arrhythmic death were similar between patients with ischemic and nonischemic HF after weighting.

### 4.1 Primary prophylactic ICD therapy in contemporary patients with HFrEF

In recent years, the long‐term prognosis of patients with HF has improved with advances in evidence‐based therapy for HFrEF, such as β‐blockers, aldosterone antagonists, CRT, and later, sacubitril/valsartan; this has led to a steady decrease in SCD rates beyond the expected reduction in the risk of HF and all‐cause mortality. In the recent DANISH trial, only 21.6% of patients in the ICD arm died after 67.7 months of median follow‐up.^10^ This trend has also been reported in contemporary cohorts of ICD^24^ and in patients with HF.^25^ However, the all‐cause mortality in our cohort (26% after a median follow‐up of 37.3 months) was similar to the mortality rate of patients randomized to the ICD arm in the MADIT‐II^1^ (14.2% after a mean follow‐up of 20 months) and SCD‐HeFT trials^16^ (22% after a median follow‐up of 45.5 months). The relatively high mortality rate in our cohort may be because of the fact that the patients included in our cohort had a higher burden of comorbidities. Additionally, the proportion of patients taking beta‐blockers, ACEI/ARBs, and aldosterone antagonists were lower in our cohort than in the DANISH trial. Especially, only half of the patients were on beta‐blockers in our cohort, while beta‐blockers were given to more than 90% of the patients in the DANISH trial. Finally, the increase in cardiovascular prevention strategies, better medical management of coronary artery disease, and the extent of coronary revascularization may also explain this phenomenon.

Patients in the nonischemic HF cohort in this study were younger than those in the DANISH study (median, 64 [IQR, 56‐72] years), and the age was similar to that in the SCD‐HeFT trial (median, 60.4 [IQR, 51.7‐68.3] years). Although the majority of deaths in people with chronic systolic HF are primarily because of cardiovascular causes such as fatal arrhythmia and worsening HF, a significant number of patients die from noncardiovascular causes.^26^ Younger patients may be more susceptible to ventricular tachycardia, while older patients are more likely to die from pump failure or noncardiovascular problems.^26^ In the post hoc analysis of the DANISH study, ICD implantation was consistently associated with a reduction in all‐cause mortality in patients aged ≤70 years. Furthermore, the benefit of ICD implantation decreased with age and was not apparent in patients aged >70 years. Older patients were more likely to die from causes other than SCD than younger patients, which might explain why the association between ICD transplantation and all‐cause death decreased with increasing age.^27^

### 4.2 Mortality in patients with nonischemic and ischemic HF who underwent prophylactic ICD therapy

In this study, the mortality rate was similar in patients with nonischemic and ischemic HF who underwent prophylactic ICD therapy after weighting. Therefore, the benefit of prophylactic ICD therapy in HF patients should not be restricted to ischemic patients. The use of prophylactic ICD therapy for the primary prevention of SCD in patients with nonischemic HF has been debated in recent years. In the SCD‐HeFT trial, ICD implantation reduced mortality by 21% and 27% in patients with ischemic HF and those with nonischemic HF, respectively.^2^ However, in the Defibrillators in Non‐Ischemic Cardiomyopathy Treatment Evaluation (DEFINITE) trial, which enrolled 458 patients with nonischemic dilated cardiomyopathy, ICDs significantly reduced the risk of SCD, but the reduction in all‐cause mortality only approximated statistical significance.^7^ In the recently performed DANISH trial, no significant effect on all‐cause mortality was observed over a median follow‐up of approximately 5 years.^10^ It is notable that the DANISH trial enrolled a large proportion of patients who received CRT (58%), which may have lowered the overall mortality as a result of disease modification.^28^ Therefore, the chance of observing any effect of ICD implantation in addition to that of CRT in the DANISH trial may have been limited a priori. Meta‐analyses incorporating data from all randomized controlled trials testing primary prophylactic ICDs, including the DANISH trial, have confirmed a significant reduction in all‐cause mortality associated with ICD use in patients with nonischemic HF.^10^, ^29^, ^30^ This may suggest that the DANISH trial was not sufficiently powered to test its primary endpoint over an extended follow‐up period, which might have led to a late alignment of the Kaplan‐Meier curves.^10^

### 4.3 Study limitations

There are several limitations to the present study. First, studies using administrative databases may be susceptible to errors arising from coding inaccuracies. To minimize this problem, we applied the definition that was validated in previous studies using the Korean NHIS sample cohort.^17^, ^18^, ^19^, ^20^ Second, although we performed adjustment by propensity score overlap weighting, residual and unmeasured confounding cannot be ruled out. Moreover, because propensity score weighting is a statistical technique that attempts to estimate the effect of a treatment, policy, or other intervention by accounting for the covariates that predict receiving the treatment, there may be methodological controversy over the use of propensity score weighting to estimate the probability of the presence of ischemic heart disease. Third, the balancing of two groups was limited by a lack of important data, such as New York Heart Association class, LVEF, and N‐terminal pro‐B‐type natriuretic peptide levels. Fourth, we had limited data on HF etiology, and thus, we can only speculate about the ischemic or nonischemic cause of HFrEF. Based on our definition of ischemic HF, we may have missed patients with ischemic HF with no history of myocardial infarction or coronary revascularization. Fifth, only half of the patients were on beta‐blockers in this study. The low beta‐blocker use rate in this study may be partly explained by the high proportion of patients with a history of COPD. However, in real clinical practice of Korea, the proportion of patients with Guideline Oriented Medicine (GDMT) may be low. Because GDMT is a prerequisite for discussing the benefits of ICD in current clinical practice, the findings of this study should be interpreted with caution. Finally, ICD is being much underutilized in Korea especially in primary prevention purpose. Thus, the study population may not represent the general ICD eligible patients in Korea. Accordingly, the study results should be interpreted with caution.

## 5 CONCLUSIONS

In contemporary HF patients undergoing prophylactic ICD implantation in Korea, all‐cause, cardiac, and noncardiac mortality rates were similar between patients with nonischemic and ischemic HF when weighting is performed to account for differences in patient characteristics. Therefore, the benefit of prophylactic ICD therapy in HF patients should not be restricted to ischemic patients. We identified a higher overall mortality in HF patients who underwent ICD implantation in Korea compared to that in recently published DANISH trial, with a lower usage of beta‐blockers, ACEI/ARBs, and aldosterone antagonists. So, our findings support the current guidelines recommendation for primary‐prevention ICD in HFrEF patients with ischemic or nonischemic HF and call for better implementation of medical therapy in clinical practice.

## CONFLICT OF INTEREST

Dr Boyoung Joung has served as a speaker for Bayer, BMS/Pfizer, Medtronic, and Daiichi‐Sankyo and received research funds from Medtronic and Abbott. No fees were directly or personally received. Younghyun Kang and Hyung‐Deuk Park are employees of Medtronic Korea. The other authors have nothing to declare.

## AUTHOR CONTRIBUTIONS

Yang PS, Kang Y., Park HD, and Joung B. designed the study, conducted data analysis, and interpretation. Yang PS and Joung B. wrote the manuscript. Bae HJ and Sung JH participated in the interpretation of data and critical revision. All authors have reviewed and approved the final version of the manuscript.

## DISCLOSURE

The protocol for this research project has been approved by a suitably constituted Ethics Committee of the institution and it conforms to the provisions of the Declaration of Helsinki. Committee of the Institutional Review Board of the CHA University Health System, Approval No. CHAMC 2018‐12‐010. As this is a retrospective study, the board waived the requirement for informed consent.

Dr Boyoung Joung has served as a speaker for Bayer, BMS/Pfizer, Medtronic, and Daiichi‐Sankyo and received research funds from Medtronic and Abbott. No fees were directly or personally received. Younghyun Kang and Hyung‐Deuk Park are employees of Medtronic Korea. The other authors have nothing to declare.

## Supporting information

Table S1‐S2
