# Supplementary material for: Mortality among ischemic and nonischemic heart failure patients with a primary implantable cardioverter‐defibrillator
Source: J Arrhythm. 2021 Oct 29;37(6):1537–45. doi: 10.1002/joa3.12651 (PMC8637096; doi:10.1002/joa3.12651)
Supplement: Supplementary file 1 — Table S1‐S2 [file JOA3-37-1537-s001.docx]

**Supplementary Materials**

**Supplementary Table 1.** Definitions and ICD-10 codes used for defining the comorbidities and clinical outcomes.

| **Comorbidities** | **Definitions** | **ICD-10 codes or conditions** |
| --- | --- | --- |
| Heart failure[^1^](#_ENREF_1) | Defined from diagnosis* | ICD-10: I11.0, I50, I97.1 |
| Myocardial infarction[^2^](#_ENREF_2) | Defined from diagnosis* | ICD-10: I21, I22, I25.2 |
| Coronary heart disease | Defined from diagnosis and intervention | Acute myocardial infarction (ICD-10: I20x, I21x) and coronary angiography (HA670, HA680, HA681) |
|  | Coronary revascularization | PTCA (Percutaneous Transluminal Coronary Angioplasty): M6551, M6552,  PCI (Percutaneous Coronary Intervention; stent insertion): M6561, M6563, M6562, M6564  Percutaneous Transluminal Coronary Atherectomy: M6571, M6572  Percutaneous Thrombus Removal, Thrombolytic Treatment: M6634  Coronary artery bypass graft: O1641, OA641, O1642, OA642, O1647, OA647 |
|  | Defined from diagnosis* | ICD-10: I25.2, I25.5, I25.6, I25.8, I25.9 |
| Hypertension[^1^](#_ENREF_1) | Defined from diagnosis* | ICD-10: I10, I11, I12, I13, I15  Treatment: antihypertensive medication |
| Diabetes mellitus[^1^](#_ENREF_1) | Defined from diagnosis* plus treatment | ICD-10: E10, E11, E12, E13, E14  Treatment: all kinds of oral antidiabetics and insulin. |
| Atrial fibrillation[^3^](#_ENREF_3) | Defined from diagnosis* | I48 |
| Peripheral arterial disease[^1^](#_ENREF_1) | Defined from diagnosis* | ICD-10: I70.0, I70.1, I70.2, I70.8, I70.9 |
| Chronic kidney disease[^1^](#_ENREF_1) | Defined from eGFR or diagnosis*  (if laboratory value was not available, diagnosis code was used) | eGFR <60mL/min per 1.73 m^2^  ICD-10: N18, N19 |
| Chronic obstructive pulmonary disease[^4^](#_ENREF_4) | Defined from diagnosis* plus treatment | ICD-10: J42, J43(except J43.0), J44  Treatment: SABA, SAMA, LABA, LAMA, ICS, ICS+LABA, or methylxanthine (>1 months). |
| Liver disease | Defined from diagnosis of chronic liver disease, cirrhosis, and hepatitis | ICD-10: B18, K70, K71, K72, K73, K74, K76.1 |
| Cancer | Defined from diagnoses of cancer (non-benign) | ICD-10: C00-C97 |
| **Outcomes** | **Definitions** | **ICD-10 codes or conditions** |
| All-cause death | Defined from data on vital status and date of death confirmed by the National Population Registry of the Korea National Statistical Office | Central registration of death conducted on the basis of the death certificates of patients |
| Cardiac death | Defined from the causes of death confirmed by the Korea National Statistical Office | ICD-10: I05, I08, I10, I11, I12, I13, I20, I21, I22, I24, I25, I26, I28, I31, I33, I34, I35, I38, I42, I46, I47, I48, I49, I50, I51, I70, I71, I77, I82 |
| Non-cardiac death | Defined from the causes of death confirmed by the Korea National Statistical Office | All deaths except cardiac death. |
| Arrhythmic death† | Defined from the causes of death confirmed by the Korea National Statistical Office | ICD-10: I46, I49.0 |

*To ensure accuracy, comorbidities were established based on one inpatient or two outpatient records of ICD-10 codes in the database. †To avoid erroneous inclusion of the patients with non-cardiac arrest, we excluded the patient with sudden arrest diagnosis accompanied by respiratory arrest (R09.0, R09.2), gastrointestinal bleeding (I85.0, K25.0, K25.4, K26.0, K26.4, K27.0, K27.4, K92.0-K92.2), brain hemorrhage (I60.x-I62.x, S06.4-S06.6), septic shock (A41.9, R57.2), pregnancy and delivery (O00-O99), diabetic ketoacidosis (E14.1), anaphylaxis (T78.2), accidents including asphyxiation, drowning, poisoning, traffic accident, fall, and suicide (T71, T75.1, T36-T65, V01-V99, W00-19, X60-X84).

**References for Supplementary Table 1**

**1.** Kim TH, Yang PS, Kim D, et al. CHA2DS2-VASc Score for Identifying Truly Low-Risk Atrial Fibrillation for Stroke: A Korean Nationwide Cohort Study. Stroke 2017;48:2984-2990.

**2.** Lee HY, Yang PS, Kim TH, et al. Atrial fibrillation and the risk of myocardial infarction: a nation-wide propensity-matched study. Sci Rep 2017;7:12716.

**3.** Kim D, Yang PS, Jang E, et al. Increasing trends in hospital care burden of atrial fibrillation in Korea, 2006 through 2015. Heart 2018.

**4.** Song S, Yang PS, Kim TH, et al. Relation of Chronic Obstructive Pulmonary Disease to Cardiovascular Disease in the General Population. Am J Cardiol 2017;120:1399-1404.

**Supplementary Table 2.** Causes of death in patients who underwent prophylactic ICD implantation.

| **Causes of death** | **Deaths in overall patients**  **(n = 287)** | **Deaths in non-ischemic HF**  **(n=151)** | **Deaths in ischemic HF**  **(n=136)** | **p-value** |
| --- | --- | --- | --- | --- |
| **Cardiac death, n (%)** | 140 (48.8%) | 74 (49.0%) | 66 (48.5%) | 0.936 |
| Sudden arrhythmic death, n (%) | 8 (2.8%) | 4 (2.6%) | 4 (3.0%) | 0.881 |
| Heart failure death, n (%) | 95 (33.1%) | 56 (37.1%) | 39 (28.7%) | 0.131 |
| Coronary death, n (%) | 27 (9.4%) | 9 (6.0%) | 18 (13.2%) | 0.035 |
| Other cardiac death, n (%) | 10 (3.5%) | 5 (3.3%) | 5 (3.7%) | 0.866 |
| **Non-cardiac death, n (%)** | 83 (28.9%) | 42 (27.8%) | 41 (30.2%) | 0.663 |
| Cancer death, n (%) | 26 (9.1%) | 17 (11.3%) | 9 (6.6%) | 0.171 |
| Cerebrovascular death, n (%) | 7 (2.4%) | 6 (4.0%) | 1 (0.7%) | 0.076 |
| Other non-cardiac deaths, n (%) | 50 (17.4%) | 19 (12.6%) | 31 (22.8%) | 0.023 |
| **Unknown, n (%)** | 64 (22.3%) | 35 (23.2%) | 29 (21.3%) | 0.706 |
